# Diversity and immune responses against *Plasmodium falciparum* gametocytes in non-febrile school children living in Southern Ghana

**DOI:** 10.1186/s12936-019-2895-7

**Published:** 2019-08-01

**Authors:** Linda E. Amoah, Hamza B. Abagna, Ruth Ayanful-Torgby, Samuel O. Blankson, Nii A. Aryee

**Affiliations:** 1 Noguchi Memorial Institute for Medical Research, College of Health Sciences, University of Ghana, Legon, Ghana; 2 West Africa Center for Cell Biology of Infectious Pathogens (WACCBIP), College of Basic and Applied Sciences, University of Ghana, Legon, Ghana; 3 Department of Medical Biochemistry, College of Health Sciences, University of Ghana, Legon, Ghana

**Keywords:** IgG responses, Gametocyte, Malaria transmission, Relative avidity, Pfs230

## Abstract

**Background:**

Natural exposure to gametocytes can result in the development of immunity against the gametocyte by the host as well as genetic diversity in the gametocyte. This study evaluated the quantity and quality of natural immune responses against a gametocyte antigen, Pfs230 as well as the prevalence and diversity of gametocytes circulating in children living in two communities in southern Ghana.

**Methods:**

Whole blood (2.5 ml) was collected from 137 non-febrile school children aged between 6 and 12 years old quarterly for a 6-month period. A drop of blood was used to prepare thick and thin blood films for parasite prevalence and density estimation. Subsequently, stored plasma samples were used in ELISAs assays to measure antibody responses and avidity against Pfs230. RNA was extraction from Trizol preserved packed cells and subsequently converted to complementary DNA (cDNA) which was used for reverse transcriptase PCR (RT-PCR) to determine gametocytes prevalence and diversity.

**Results:**

Gametocyte carriage in the peak season (July) determined by *Pfg377* RT-PCR was 49.2% in Obom and 22.2% in Abura, and was higher than that determined by microscopy. Gametocyte diversity was low and predominated by the same allele at both sites. The relative avidity index for antibodies measured in Abura was higher than that recorded in Obom at all time points although Pfs230 IgG concentrations were significantly high (P < 0.0001) in Obom than in Abura at all time points. The IgG responses in Obom were significantly higher than that in Abura during the peak season.

**Conclusion:**

Naturally induced antibody responses against Pfs230 in children living in both high perennial and low seasonal malaria transmission settings reduced significantly in moving from the peak to the off-peak season. The relative avidity of antibodies against Pfs230 in Abura was significantly higher than those measured in Obom, despite having lower IgG levels. Very limited diversity was identified in the gametocytes circulating in both Obom and Abura.

**Electronic supplementary material:**

The online version of this article (10.1186/s12936-019-2895-7) contains supplementary material, which is available to authorized users.

## Background

Malaria associated morbidity and mortality in Ghana has steadily declined [1, 2]. This is attributed to numerous control interventions targeting vectors and parasites [1]. Despite all these interventions, the high rate of asymptomatic *Plasmodium* parasite carriage is likely to enhance malaria transmission [3, 4]. During *Plasmodium falciparum* infections, the human host develops immune responses against exposed parasite antigens, which increases after repeated exposure. These immune responses are directed against both the asexual as well as the sexual (gametocyte) transmissible forms of the parasite [5–7]. Gametocyte carriage in asymptomatic infections is prevalent, however mostly at low (submicroscopic) densities [3, 8] and is generally more prevalent in children than in adults [9]. Though microscopy is the gold standard for parasite detection, the low densities of the gametocytes warrants more sensitive molecular tools including reverse transcriptase (RT) or real time reverse transcriptase (q) polymerase chain reaction (PCR) to make measurements more accurate [8, 9].

Genetic diversity in the malaria parasite is a major set back to the design of an effective malaria vaccine [10, 11]. The gametocyte-specific protein Pfg377, which is associated with osmiophilic bodies in *P. falciparum* is a single copy intron-less gene with 4 repetitive regions, R1–R4 [8, 12]. Genetic diversity of *P. falciparum* gametocytes has been efficiently determined by genotyping region 3, the most polymorphic region of Pfg377 [8, 12].

Antibodies against some gametocyte antigens, including Pfs230 and Pfs48/45 have been shown to reduce malaria transmission due to their possession of transmission reducing activities [13]. Seasonal variations in the prevalence and diversity of gametocytes have the potential to influence malaria transmission as well as the quality and quantity of naturally induced antibodies against these gametocytes in the human hosts.

Malaria transmission patterns vary across Ghana with some communities exhibiting perennial malaria transmission and others seasonal malaria transmission. This study sought to determine whether gametocyte prevalence or diversity, and/or naturally-induced immune responses against gametocyte antigens could be used to explain the malaria transmission patterns observed in the selected study sites. As such, the prevalence and diversity of gametocytes as well as the quantity and quality of naturally induced antibody (IgG) responses to the gametocyte antigen Pfs230 in children living in an area of perennial malaria transmission and another area with seasonal malaria transmission were determined.

## Methods

### Ethical statement

Approval for the study was obtained from Noguchi Memorial Institute for Medical Research (NMIMR) IRB. Permission was also sought from Ghana Education Service and written consent were obtained from parents or guardians prior to recruitment as stated and described in previous studies [5, 14].

### Study site and sample collection

The longitudinal study enrolled school children aged between 6 and 12 years old, living in Obom and Abura in southern Ghana. Characteristic features of Obom, a town with high and perennial malaria transmission and Abura, a town with low and seasonal malaria transmission have been previously described [5]. This study used samples from 65 children from Obom and 72 children from Abura, who were present during the July 2015 (Peak season), October 2015 (end of the peak season) and January 2016 (off peak season) sample collection time points. At each time point, 2.5 ml of venous blood was collected into EDTA vacutainer tubes. A drop of blood (about 10 µl) was used to prepare thick and thin blood smears, after which the blood was separated via centrifugation into plasma and packed cells. The plasma was collected into a 1.5 ml Eppendorf tube and used for the IgG and relative avidity ELISAs. An aliquot (50 µl) of the packed cells were stored in 250 µl of Trizol reagent (Invitrogen, USA) and used for RNA extraction and subsequent cDNA preparation.

### Microscopic examination of blood smears

Staining of the thick and thin blood smears were as previously described [6] and according to World Health Organization (WHO) guidelines [15]. Parasite density (PD) was estimated based on visual counting of the number of infected red blood cells counted per 200 white blood cells, as recommended by the WHO [16]. Two independent microscopists read each slide.

### Molecular assays

#### Extraction of RNA and cDNA preparation

RNA extraction was done using the Quick RNA miniprep kit (Zymo Research, USA) that included an on-column DNase 1 treatment prior to elution according to manufacturer’s instructions as previously reported [17]. The purity and concentration of the extracted RNA was checked using a Nanodrop 2000c spectrophotometer (Thermo Fisher Scientific, USA).

Extracted RNA was converted into cDNA using the Protoscript II first strand cDNA synthesis kit (NEB, UK) according to manufacturer’s instructions. Briefly, a mix of 2.5 µM oligo dT (NEB, UK) and 3.0 µM Random primer mix (NEB, UK) were added to 6 µl of the extracted RNA and incubated at 65 °C for 5 min. Subsequently, 10 µl of 1× Protoscript II reaction mix and 2 µl of 1× Protoscript II enzyme mix were added to the mixture and incubated for 60 min at 42 °C and then for 5 min at 80 °C. The resulting cDNA was stored at − 20 °C until further use.

#### *Pfg377* genotyping

Gametocyte diversity was assessed at the Pfg377 locus in genomic DNA (gDNA), cDNA and RNA samples using a previously published protocol [8]. The nested PCR of the *Pfg*377 gene was performed using the GOTaq hot start master mix (Promega Corporation, USA). The amplification for both primary and secondary PCR reactions contained 1× GoTaq master mix and 20 µM of the primers (Additional file 1). The volume of template used for gametocyte diversity was 1 µl of cDNA, whilst 3 µl of RNA was used to check for gDNA contamination of the RNA samples. Nuclease free water template was used as negative control and each sample corresponding gDNA as well as cDNA from laboratory cultured gametocytes from the 3D7 *P. falciparum* parasite isolate were used as positive controls for the reactions. The reactions cycling conditions for both RNA, DNA and cDNA comprised of an initial denaturation at 94 °C for 2 min, 30 cycles (94 °C for 30 s, 55 °C for 30 s, 72 °C for 30 s), 72 °C for 5 min, and then 4 °C on hold.

The amplified PCR products were loaded onto a 2% agarose gel stained with ethidium bromide and electrophoresed for 30 min at 100 V. Resolved amplicons were visualized using a UV trans-illuminator (Vilber, Germany). The absence of amplified PCR products on the gel in the lanes containing RNA template serve as an indication that gDNA was not carried over during the RNA extraction and purification processes.

### Immunological assays

Naturally induced antibody responses against *P. falciparum* gametocyte antigen Pfs230 in the plasma collected from children were determined using ELISA as previously described [5, 6]. Plasma samples that had previously been identified as containing high levels of Pfs230 [5, 6] were pooled and used as a positive control. Plasma from malaria naïve individuals from Denmark served as negative control samples. Recombinant IgG, (BP055, the Binding Site) was used as the standard calibrator for total IgG measurements. The Pfs230 antigen was diluted to 1 µg/ml in carbonate buffer, pH 9.0 and coated at 100 µl/well onto ELISA plates at 4 °C overnight. The measured OD values were converted into concentration using the ADAMSEL software (Ed Remarque).

#### Avidity ELISA

The relative avidity of IgG responses to Pfs230 was determined as previously reported [18]. The procedure was similar to the standard ELISA protocol detailed above, with the exception of an additional step where 100 µl of 2.4 M sodium thiocyanate (NaSCN) was add to the plate and incubated for 10 min. The plates were subsequently washed 4 times with wash buffer and then incubated with the secondary antibody. The proceeding processes were identical to that of the ELISA described above.

### Data analysis

The number of infected red blood cells observed per 200 white blood cells (WBC) counted on the thick smear was recorded and converted into parasite density (PD) by multiplying the number by 40, based on the assumption of 8000 WBCs μl per blood.

ELISA data was analysed using the ADAMSEL software, which converts optical density (OD) values into weighed concentrations (wConcs), which represents the antibody (IgG) responses to the antigen. Graphs were plotted using GraphPad Prism version 5. Kruskal–Wallis test (GraphPad prism) was used to determine whether there were any statistically significant differences between the parasite density measured across the towns and between visits. This was followed by the Dunn’s multiple comparison test (a post hoc test), to find which specific means are significant from others (p < 0.05). Column statistics (GraphPad prism) was used to determine the medians and inter-quartile ranges of the wConcs (IgG levels) in order to determine the measure of dispersion. The mean wConcs (Antibody (IgG) responses) were compared between the sites using nonparametric Mann–Whitney U test (paired two tailed t-test). Independent samples T-test was used when comparing the ages of the children, PD and gametocyte prevalence. *P*-*values* less than 0.05 were considered significant.

The relative avidity index (RAI) for anti-Pfs230 IgG was calculated as the ratio of the OD of the sodium thiocyanate-treated sample (numerator) to the OD of the untreated sample (denominator) multiplied by 100. (RAI = [Sodium thiocyanate (NaSCN) treated IgG/untreated IgG] × 100).

## Results

Only samples collected from 65 children living in Obom and 72 children living in Abura who were present for all three sample collections (July 2015, October 2015 and January 2016) were analysed in this study. There was no statistical difference (corrected p = 0.763) between the ages of the children from both sites (Table 1). The prevalence of female children was similar in both sites and only slightly higher than the male children (Table 1). Parasite density (PD) in Obom in October and January were similar (corrected p = ns) and both significantly lower than in July [corrected p < 0.001 (October/July) and 0.05 (January/July)]. A similar trend in PD was observed in Abura where PD for October and January were similar (corrected p = ns) but both significantly lower that that determined in July (corrected p < 0.001 for both October/July and January/July).

**Table 1:** Demographic data of study participants

	Obom (N = 65)	Abura (N = 72)
Age (years)		
Median	9	9
Range (min–max)	6–12	6–11
Females (%)	31 (53)	40 (57)
Parasite density (P/μl)		
July 2015		
Count	44	41
Median (n)	5426	4500
Min–max	38–32,609	148–16,759
October 2015		
Count	16	13
Median (n)	1546	1065
Min–max	298–6170	325–4000
January 2016		
Count	8	22
Median (n)	1904	1470
Min–max	600–4000	120–8200

Min, minimum; max, maximum; P/μl, asexual parasites per μl blood; n, number of children that were parasite positive (asymptomatic)

### Gametocyte prevalence and diversity

Microscopic evaluation of thick blood smears identified a very low prevalence of gametocytes (Table 2). Gametocyte prevalence estimated by microscopy was higher in Abura in July and October than in Obom, and reduced over the course of the study in both sites (Table 2). Gametocyte prevalence increased significantly in both sites (corrected = 0.0029 (Obom); p < 0.0001 (Abura)) when Pfg377 RT-PCR was used to detect total (microscopic and submicroscopic) gametocyte prevalence (Table 2). Submicroscopic gametocyte prevalence was significantly higher in Obom than in Abura in July (corrected p < 0.0001) during the peak season. No Pfg377 RT-PCR measurements were performed in October and January (Table 2). Gametocyte prevalence (both microscopic and submicroscopic) in the peak season did not correlate with asexual parasite density in either of the two study sites (p > 0.05 for all comparisons).

**Table 2:** Estimation of gametocyte prevalence

Site	Month	RT-PCR (%)	Micro (%)
Obom	Jul-15	32 (49.2)	2 (3.1)
	Oct-15	ND	0
	Jan-16	ND	0
Abura	Jul-15	16 (22.2)	6 (8.3)
	Oct-15	ND	1 (1.4)
	Jan-16	ND	0

RT-PCR, positive by Pfg377 RT-PCR; Micro, microscopy positive; ND, not determined; Jul-15, July 2015; Oct-15, October 2015; Jan-16, January 2016

**Fig. 1:**
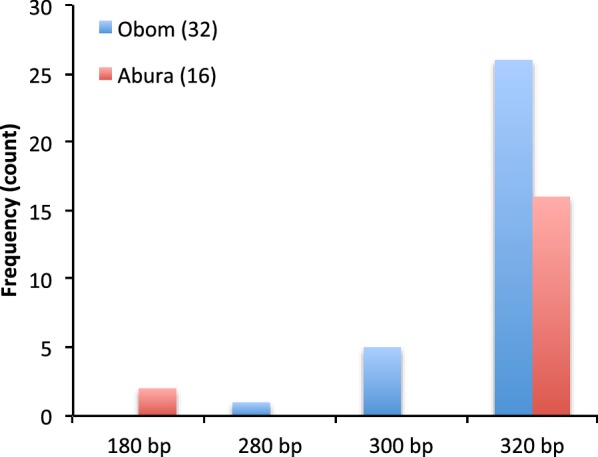
Gametocyte diversity. The graph illustrates the frequency at which each of the various Pfg377 alleles were identified at each of the study sites. The number in brackets represents the total number of samples. Obom (blue) and Abura (orange)

Diversity in the Pfg377 gene in the parasite isolates was grouped according to band size, which varied between 180 bp to 320 bp (Fig. 1). Although distinct alleles were found specifically in all study sites, the 180 bp allele was only found in Abura, whereas the 280 and 300 alleles were observed only in Obom. The 320 bp allele was the most prevalent variant in both sites (Fig. 1). All samples identified in Obom were clonal (contained a single clone) as opposed to two samples in Abura that contained two different clones.

### Naturally induced antibody (IgG) responses

**Fig. 2:**
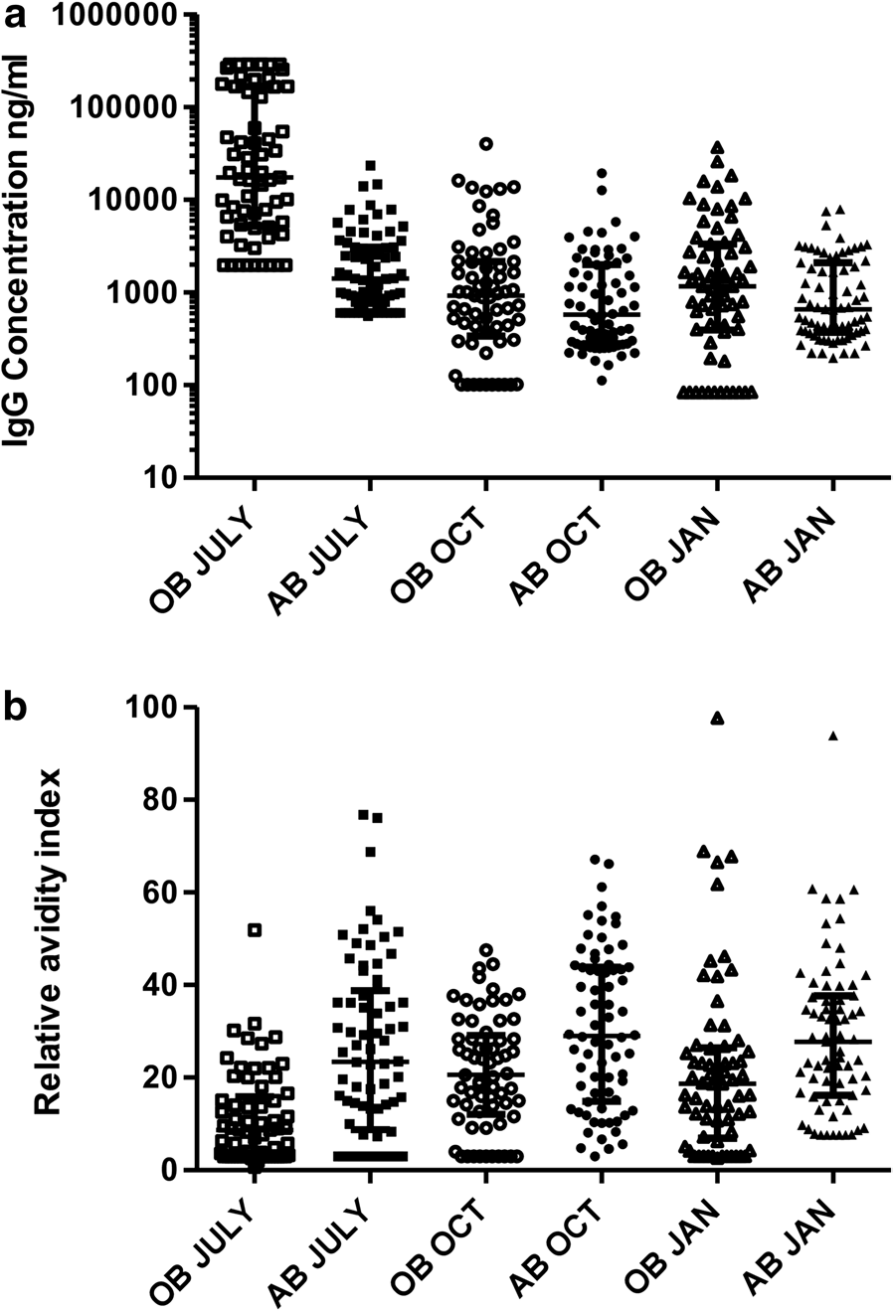
Naturally induced antibody responses against Pfs230. **a** Antibody concentrations and **b** Relative avidity (RAI) of IgG. *OB* Obom, *AB* Abura. The IgG concentrations are reported as the log10 of the median values, with the error presented by the interquartile range. The RAI graph is presented as the median with the interquartile range representing the error. Obom, open and Abura, solid symbols (square, July; circle, October and triangle, January)

The mean wConc of IgG responses against Pfs230 in Obom decreased significantly in moving from the peak (July) to the off peak season (corrected p < 0.001 for both July & October and July & January). No significant difference was observed in the mean IgG responses recoded in October and January (Fig. 2a). The mean wConc of IgG responses in Abura exhibited a similar pattern, with IgG responses in July being significantly higher than those measured in both October and January [corrected p < 0.01 (July & October) and p < 0.05 (July and January)].

The mean wConc of IgG responses in Obom were significantly higher than those measured in Abura during the peak season (corrected p < 0.001) (Fig. 2a). In October and January, antibody responses against Pfs230 measured in children were similar at both sites (Fig. 2).

The relative avidity index (RAI) of antibody responses (Pfs230 IgG) in Obom was significantly lower than those measured in Abura at all the measured time points (corrected p < 0.0001,0.01 and 0.01, respectively) (Fig. 2b). The relative avidity of antibody responses against Pfs230 in Abura was similar over the course of the study (corrected p > 0.05). In Obom, the relative avidity of antibody responses against Pfs230 was significantly lower in July (corrected p < 0.001 (October) and 0.01 (January) relative to those measured during October and January, which were comparable (corrected p = 0.885).

## Discussion

Most immunological surveys that try to understand and characterize malaria transmission use tools including the measurement of antibodies against asexual parasites [19] and sporozoite [20] as well as asexual parasite density [21] and do not usually include gametocyte density and immune responses against gametocytes [22], which are the transmissible forms of the parasite. Diversity in asexual parasite antigens has been found to be a major contributor to the acquisition antibodies with increasing breadth [23] and ability to target multiple parasite strains. Similarly, exposure to genetically diverse gametocytes may contribute to the acquisition of high quality functional anti-gametocyte antibodies. Although there is a large pool of information on *P. falciparum* parasite diversity and its role in the development of anti-parasite immunity, very little information is available on the diversity of *P. falciparum* gametocytes as well as the quality of naturally induced antibody responses against gametocyte antigens. In this study gametocytes circulating in two communities in southern Ghana were genotyped at the highly polymorphic region 3 of Pfg377, to determine their diversity. Variations in the quantity and relative avidity of naturally induced antibodies against the gametocyte antigen Pfs230 during the peak and off peak malaria season were also determined.

The significantly higher prevalence of submicroscopic gametocytes in the high transmission setting relative to the low transmission setting in the peak season despite the similar prevalence of microscopic density gametocytes could be due to the fact that majority of gametocytes contained in infections are at submicroscopic densities [24]. This finding is similar to a previous report in a smaller number of children from the same communities [3].

The limited diversity in the sequence of the gametocyte antigen Pfg377 identified in this study prevents its use to estimate variations in malaria transmission intensity. The limited diversity observed supports an earlier report from southern Ghana where limited diversity was identified in gametocyte antigens Pfs48/45 and Pfs230 [6]. The largest sized allele was the most prevalent in both communities, which supports the notion that gametocyte diversity is limited. The absence of any significant difference in gametocyte diversity observed in this study could be due to the use of PCR genotyping as opposed to nucleic acid sequencing or fragment analysis. The observed diversity could have been enhanced if the PCR products were resolved using a technique such as capillary electrophoresis. Diversity in gametocyte antigens have however been suggested to be much lower than diversity in the asexual parasite [6, 25].

The naturally induced immune responses against Pfs230 were found to be significantly higher in Obom (high transmission setting) than in Abura (low transmission setting) only during the peak season, most likely due to increased exposure to high-density gametocyte infections during the peak season relative to the other seasons. These results are contrary to an earlier report on a larger number of school children from the same study site, where anti-Pfs230 IgG responses in children measured in January were significantly higher in children from Obom than children from Abura [5]. The similar trends observed in antibody responses in Obom and Abura over the course of this study suggests antibody responses against Pfs230 is enhanced by the presence of active infections containing gametocytes, since the concentrations reduced with a reduction in gametocyte prevalence. Similar findings have been reported where naturally induced antibody responses against sexual stage antigens including Pfs230 were found to be relatively short lived and persist for less than 3 months [26].

The quality of antibody responses against Pfs230 assessed by relative avidity was significantly lower in Obom than in Abura despite the higher antibody levels measured could be attributed to the slightly lower gametocyte diversity that was identified in Abura relative to Obom. These results are similar to a previous report, which identified the relative avidity of antibody responses against EBA 175 to be lower in a community with high antibody concentrations and diversity than those measured in a community with lower antibody concentrations and diversity [18].

These results suggest that high transmission intensity settings are associated with higher levels of low avidity antibodies relative to low transmission settings, which are associated with low levels of high avidity antibodies. The observed similarity in the RAI of IgG against Pfs230 over the course of the changing malaria seasons in Abura and the variability observed in Obom could not be related to gametocyte diversity or gametocyte prevalence because Pfg377 measurements were only performed during the peak season. However, in this study the molecular estimate of gametocyte prevalence was similar despite significant differences between IgG and RAI of antibodies against Pfs230 in Obom and Abura. This suggests that factors other than concurrent gametocyte prevalence maybe needed to explain Pfs230 IgG and avidity levels. Similarly, although antigen diversity is expected to influence antibody avidity [27], the low diversity in gametocytes identified in this study prevented its use in explaining the differences observed in antibody avidity in the high and low transmission setting.

More details on the genetic diversity of gametocytes circulating in different seasons at high and low malaria transmission settings is required to enhance our understanding of malaria transmission. A larger sample size as well as the use of a genotyping tool with higher sensitivity such as nucleic acid sequencing might have increased the observed diversity identified in the gametocytes circulating in the two study sites.

## Conclusion

Naturally induced antibody responses against Pfs230 in children living in both high perennial (Obom) and low seasonal (Abura) malaria transmission settings reduced significantly in moving from the peak to the off-peak season. The relative avidity of antibodies against Pfs230 in Abura was significantly higher than those measured in Obom, despite having lower IgG levels. Very limited diversity was identified in the gametocytes circulating in both Obom and Abura.

## Additional file

**Additional file 1.** Gametocyte (Pfg377) genotyping primers.

## Data Availability

All data generated or analyzed during this study are included in this published article (and its additional file).

## Abbreviations

RAI: relative avidity index
bp: base pair
RNA: ribonucleic acid
DNA: deoxyribonucleic acid
cDNA: complementary deoxyribonucleic acid
mRNA: messenger ribonucleic acid
RT-PCR: reverse transcriptase-polymerase chain reaction
OD: optical density
wConc: weighted concentration
IgG: immunoglobulin G
PD: parasite density
