# Supplementary material for: Diversity and immune responses against Plasmodium falciparum gametocytes in non-febrile school children living in Southern Ghana
Source: Malar J. 2019 Aug 1;18:265. doi: 10.1186/s12936-019-2895-7 (PMC6676606; doi:10.1186/s12936-019-2895-7)
Supplement: Supplementary file 1 — Additional file 1. Gametocyte (Pfg377) genotyping primers. [file 12936_2019_2895_MOESM1_ESM.docx]

Additional file 1. Gametocyte (Pfg377) genotyping primers

| *Pfg377* |  |
| --- | --- |
| **Outer** | R3D1: GATGAAGGGATATATCACCTCACAATGTG |
|  | R3R2: GTCATGATTTTCTTCTCCTTCGGATATGG |
| **Inner** | R3D2: CCATAGGAATATTACACCATATCATGTG |
|  | R3R1: TATGGTGATAAATGAGGAGTGTCCCCTTAC |

Primer sequences for the PCR amplifications were obtained from Menegon, M *et al^9^*
